# ASCE-PPIS: a protein–protein interaction sites predictor based on equivariant graph neural network with fusion of structure-aware pooling and graph collapse

**DOI:** 10.1093/bioinformatics/btaf423

**Published:** 2025-07-24

**Authors:** Guanghao Shen, Ziqi Zhang, Zhaohong Deng, Xiaoyong Pan, Hong-Bin Shen, Dong-Jun Yu, Shudong Hu, Yuxi Ge

**Affiliations:** School of Artificial Intelligence and Computer Science, Jiangnan University, Wuxi, Jiangsu 214012, China; Engineering Research Center of Intelligent Technology for Healthcare, Ministry of Education, Jiangnan University, Wuxi, Jiangsu 214012, China; School of Artificial Intelligence and Computer Science, Jiangnan University, Wuxi, Jiangsu 214012, China; Engineering Research Center of Intelligent Technology for Healthcare, Ministry of Education, Jiangnan University, Wuxi, Jiangsu 214012, China; School of Artificial Intelligence and Computer Science, Jiangnan University, Wuxi, Jiangsu 214012, China; Engineering Research Center of Intelligent Technology for Healthcare, Ministry of Education, Jiangnan University, Wuxi, Jiangsu 214012, China; School of Automation and Intelligent Sensing, Shanghai Jiao Tong University, Shanghai 200240, China; School of Automation and Intelligent Sensing, Shanghai Jiao Tong University, Shanghai 200240, China; School of Computer Science and Engineering, Nanjing University of Science and Technology, Nanjing 210014, China China; Affiliated Hospital of Jiangnan University, Wuxi 214000, China; Affiliated Hospital of Jiangnan University, Wuxi 214000, China

## Abstract

**Motivation:**

Identifying protein–protein interaction sites constitute a crucial step in understanding disease mechanisms and drug development. As experimental methods for PPIS identification are expensive and time-consuming, numerous computational screening approaches have been developed, among which graph neural network-based methods have achieved remarkable progress in recent years. However, existing methods lack the utilization of interactions between amino acid molecules and fail to address the dense characteristics of protein graphs.

**Results:**

We propose ASCE-PPIS, an equivariant graph neural network-based method for protein–protein interaction prediction. This novel approach integrates graph pooling and graph collapse to address the aforementioned challenges. Our model learns molecular features and interactions through an equivariant neural network, and constructs subgraphs to acquire multi-scale features based on a structure-adaptive sampling strategy, and fuses the information of the original and subgraphs through graph collapse. Finally, we fusing protein large language model features through the ensemble strategy based on bagging and meta-modeling to improve the generalization performance on different proteins. Experimental results demonstrate that ASCE-PPIS achieves over 10% performance improvement compared to existing methods on the Test60 dataset, highlighting its potential in PPI site prediction tasks.

**Availability and implementation:**

The datasets and the source codes along with the pre-trained models of ASCE-PPIS are available at https://github.com/nunhehheh/ASCE-PPIS.

## 1 Introduction

Protein–protein interaction site (PPIS) prediction holds pivotal significance in life sciences and drug design. PPIS serve as the foundation for biological processes such as intracellular signal transduction and metabolic regulation. Aberrant PPIS have been implicated in the pathogenesis of various diseases, including cancer and autoimmune disorders (Zhang and Kurgan 2018). Therefore, developing robust predictive methods is critical for elucidating protein functions, identifying druggable targets, and advancing innovative therapeutic strategies.

Currently, methods for identifying PPIS can be categorized into two groups: traditional wet experimental approaches and machine learning based computational methods. However, conventional wet approaches for identifying protein interaction sites—such as X-ray crystallography and yeast two-hybrid screening—while providing invaluable experimental data for biological research, face limitations in broad applicability due to prohibitive costs and time-intensive workflows (Shoemaker and Panchenko 2007, Hamp and Rost 2015). Consequently, machine learning-based approaches have emerged as a prominent research focus in deciphering protein functions, offering a transformative pathway to overcome the limitations of conventional experimental paradigms.

Existing machine learning methods for predicting PPIS include traditional machine learning algorithms and deep learning algorithms. Early PPIS prediction methods based on traditional machine learning algorithms include Naive Bayes classifier (Murakami and Mizuguchi 2010), Random Forest (Northey *et al.* 2018), XGBoost (Deng *et al.* 2020), Support Vector Machine (Wang *et al.* 2021), logistic regression (Zhang *et al.* 2019), and so on. Although traditional machine learning methods have made certain progress in the early research of PPIS prediction due to their interpretability and ability to integrate domain knowledge, as proteomics data grows and the complexity of interactions continues to increase, these methods have gradually revealed their limitations: Firstly, the modeling approach based on handcrafted feature heavily relies on the researcher’s prior knowledge, making it challenging to capture the hidden topological correlations and long-range dependencies within amino acid sequences. Secondly, the structural limitations of shallow models prevent them from effectively addressing nonlinear mapping problems in high-dimensional sequence spaces. These shortcomings have led researchers to explore more expressive modeling paradigms. In recent years, deep learning algorithms have prompted more and more researchers to start using them due to their powerful feature abstraction capabilities. For example, ConvsPPIS (Zhu *et al.* 2020) was the first to introduce CNNs into the PPIS prediction task, and successfully extracted the deeper information of the sequences through sliding convolution; DELPHI (Li *et al.* 2021) proposed an integrated structure of CNN and RNN for effective deep mining of protein sequences; DeepPPISP (Zeng *et al.* 2020) developed a CNN-based structure to achieve the combination of global and local information about the sequence to enhance the representation of the model; Xie *et al.* (2020) proposed a data enhancement method based on CNN with statistical residue binding propensity; DLPred (Zhang *et al.* 2019) proposed a simplified LSTM model combined with multi-task learning to effectively deal with the PPIS prediction sample imbalance problem; MaSIF-site (Gainza *et al.* 2020) proposed the use of a deep learning framework, MaSIF, to extract protein molecular surface fingerprints to assist deep learning models for PPIS prediction. EnsemPPIS (Mou *et al.* 2023) proposed an ensemble framework based on transformer and gated convolutional networks, which effectively capture global and local patterns.

Despite demonstrating superior predictive performance over conventional machine learning methods, current deep learning approaches for protein–protein interaction site prediction still exhibit limited incorporation of three-dimensional (3D) protein structural information within their architectures. These restrictions arise from the traditional neural networks’ reliance on gridded data, leading to the compression of spatial structural features into low-dimensional vectors. To this end, researchers have begun to look to graph neural networks, whose unstructured data processing capabilities form a natural fit with the topological properties of proteins. Abstracting amino acids as nodes and modeling intermolecular forces, Euclidean distances, and other information as edges, this migration from sequence space to topological space allows for a fuller synthesis of sequence and structural information about proteins. For example, Graph-PPIS (Yuan *et al.* 2021) utilizes the GCNII (Chen *et al.* 2020) framework for PPIS prediction and introduces the position-specific scoring matrix (PSSM) and the definition of protein secondary structure (DSSP) as node features to apply graph deep learning to the PPIS prediction. Although GCNII is able to take into account both sequence and structure information compared to deep learning methods based on sequence learning, it is unable to identify the importance of neighboring nodes and has a limited ability to learn protein structure. Another representative approach in this area is AGAT-PPIS (Zhou *et al.* 2023), which integrates more protein structure information by improving GAT and updating node embeddings using weighted neighborhood node features and neighborhood edge features, and additionally proposes to add atomic-level features to node embeddings and learn distribution information in the scattering space through relative position embedding. Compared to Graph-PPIS, AGAT-PPIS effectively improves performance, but its time cost is higher. In order to reduce the time consumption, the GHGPR-PPIS (Zeng *et al.* 2024) method has been proposed, which combines the heat kernel and generalized PageRank techniques as well as the edge self-attentive feature processing module. Compared to AGAT-PPIS, GHGPR-PPIS improves performance while achieving a lightweight network. Another graph-based framework is Graph-Transformer. Compared to GNN, its global self-attention mechanism enables it to better handle large graphs. For example, AGF-PPIS (Fu *et al.* 2024) integrates a GCN within the Graph-Transformer framework to incorporate local structural information, achieving superior performance compared to standard Transformers.

**Figure 1.**
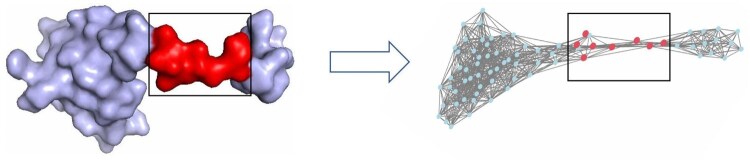
Protein 4nqw chain B, the area within the box indicates the low curvature portion; such a structure tends to impede long-distance message transmission in GNN.

The current study shows that among the existing methods for protein interaction site prediction based on machine learning and deep learning, the methods developed based on GNN show more powerful learning ability and potential. However, these methods are currently facing some key challenges:

1. Existing GNN-based PPIS prediction methods lack the utilization of vector features. The work of Schütt *et al.* (2021) demonstrates that by introducing vector features that are equivariant to translation and rotation, the model can explicitly learn information about directionally sensitive intermolecular interaction patterns, such as the vector representations of dipole moments and bond angle vibration modes. Due to the requirement for invariance to translation and rotation in the PPIS prediction task, existing GNNs can only convert equivariant 3D coordinates into scalars, such as pseudo-position embeddings and dihedral angles. Although these static attributes can describe the structural properties of nodes, they have significant limitations in describing directional interactions between amino acids. To address this, E(Q)AGNN-PPIS (Suvvada *et al.* 2025) proposes a PPIS prediction method based on the equivariant model GVPGNN, thereby avoiding the loss incurred from compressing 3D coordinate information into scalars. However, the update of vector features is independent of scalars in GVP, which prevents the integration of vectors and scalars.
2. Existing GNN-based PPIS prediction methods lack the exploitation of multi-scale features. Multi-scale features can enhance model representation and mitigate the over-smoothing problem specific to graph deep learning (Abu-El-Haija *et al.* 2019). However, due to the uniqueness of the topology, GNNs are unable to extract multiscale information as CNNs do with only different-sized convolutional kernels. To address this problem, Ding *et al.* (2024) proposed the MEG-PPIS based on Graph U-net (Gao and Ji 2019), which simulates the maximum pooling in CNN by the strategy of sampling key nodes through projection vectors, thus obtaining the features with a larger receptive field. However, the sampling strategy based on projection vectors is susceptible to the clustering effect on proteins, which tends to concentrate the sampling points in a certain neighborhood and loses the effect of enlarging the receptive field.
3. The GNN models in existing methods are prone to the failure of long-distance message passing at higher levels. As shown in Fig. 1, messages from a large number of nodes are compressed into a small number of fixed-length vectors, resulting in the negative curvature portion becoming a bottleneck in the flow of information (Topping *et al.* 2022) and hindering message delivery over long distances. This is due to the fact that protein graphs tend to exhibit densities that require the representation of exponentially large numbers of neighboring nodes to be compressed into fixed-size vectors as the felt-wild of a single node grows exponentially with the number of layers, resulting in some of the nodes becoming bottlenecks for message passing, a phenomenon known as over-squashing (Alon and Yahav 2021). Although SP-MPNN (Abboud *et al.* 2022) provides a way to optimize the topology by shortest path neighborhood for this problem, the extra edges added in this method also lead to noise.

To address the above challenges, this paper proposes a new protein interaction site predictor, ASCE-PPIS, based on structure-aware pooling and graph collapse fusion equivariant graph neural networks. First, we choose Equivariant Graph Convolutional Layer (EGCL) from Equivariant Graph Neural Network (EGNN) (Satorras *et al.* 2021) as the hidden layer to construct an orientation-sensitive vector messaging mechanism by dynamically updating the nodes’ 3D coordinates so that the model learns the vector features. In addition, we propose a strategy based on structure-aware pooling (Ranjan *et al.* 2020) for subgraph sampling from graphs, which samples nodes from the overall view of proteins through soft clustering and attention, avoiding the problem of over-sampling of local regions that is commonly found in traditional sampling methods, and thus improving the quality of multi-scale features. Furthermore, inspired by the work of Sestak *et al.* (2023) and Ying *et al.* (2018), we propose to achieve long-distance message passing by graph collapsing and unpooling, which allows each node to indirectly communicate with a distant node once by performing one message passing between each local composition on the protein. Finally, we fused amino acid representations generated by the protein large language model (LLM) into the model through an ensemble strategy based on bagging and meta-modelling, which improves the capacity of the model across different proteins. The research results indicate that ASCE-PPIS has achieved more than a 10% performance improvement over existing methods on the Test60 dataset, demonstrating its potential in the task of PPIS identification.

## 2 Materials and methods

### 2.1 Datasets

Our experiments follow the datasets in GHGPR-PPIS, including the training set Train334, three independent test sets Test60, Test315-28, and UBtest31-6, and the parameters of each dataset are shown in Table 1, where Test60 is mainly used as a benchmark test set for performance comparison, while the rest of the dataset is used to evaluate the generalization ability of the model. Each sample in each dataset consists of a protein sequence and a tag sequence, where each letter in the protein sequence indicates an amino acid and the corresponding position in the tag sequence indicates whether the amino acid is an interaction site.

**Table 1.** The statistics of the datasets.

Dataset name	Number of proteins	Number of interacting residues	Number of non-interacting residues
Train334	334	10 336	55 872
Test60	60	2075	11 069
Test315-28	287	8566	51 810
UBtest31-6	25	711	5206

### 2.2 Model architecture

**Figure 2.**
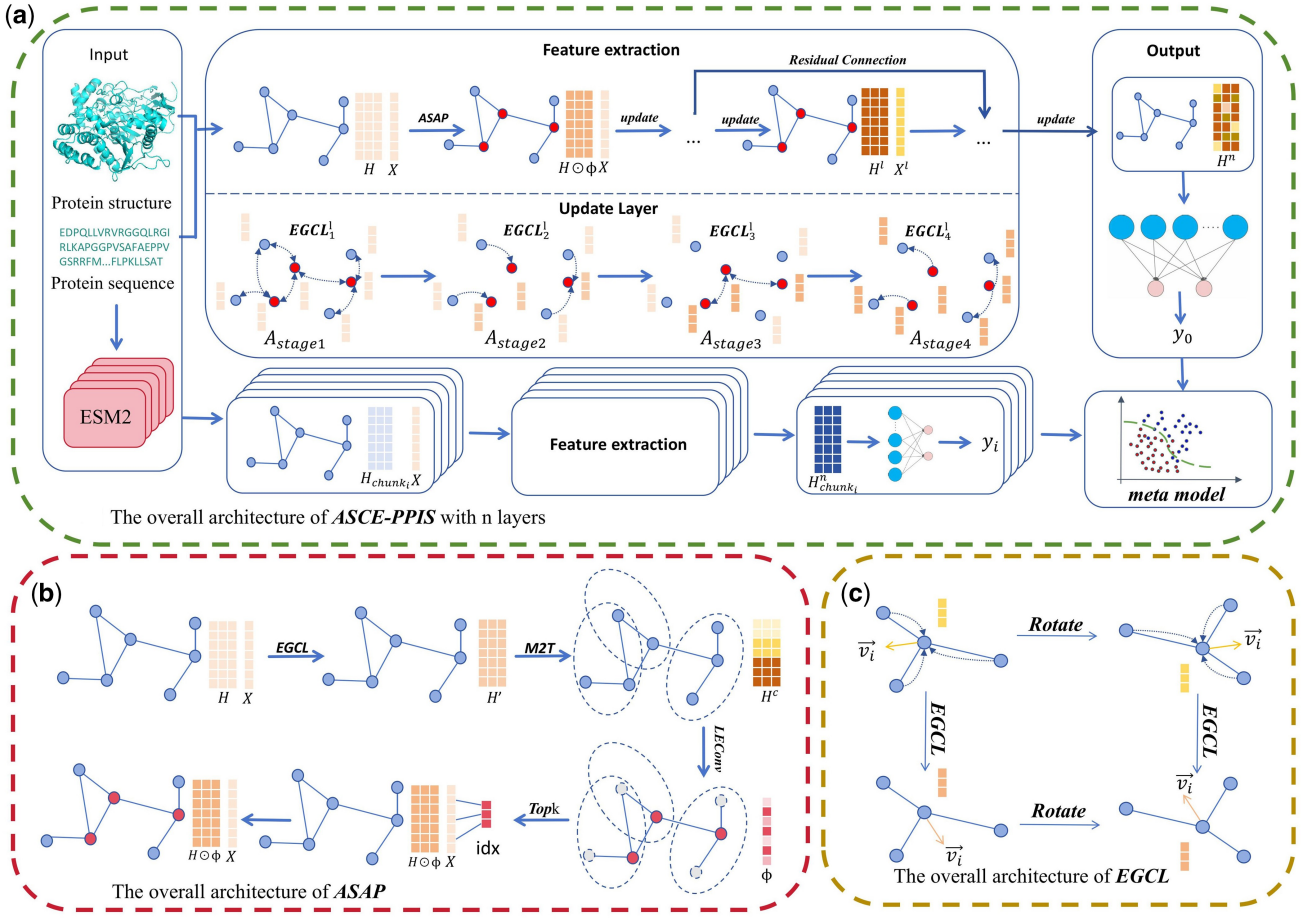
Overview of the framework of ASCE-PPIS. (a) The input module processes the sequences and 3D structures into adjacency matrices and node embeddings. In each model, the ASAP module extracts subgraphs from the original graph, messages are passed between the original and subgraphs in four steps, and finally, the output module performs a binary classification by an MLP, combining the results of multiple classifications, and finally, the ensemble model determines whether an amino acid residue is an interaction site. (b) ASAP module, samples nodes locally from a global perspective to construct subgraphs through the M2T attention mechanism. (c) Overview of the framework of EGCL, vector features will maintain equivariance during the update process.

The overall framework of the model is shown in Fig. 2a, first, we processed the protein 3D structure and sequence in the dataset into an undirected graph adjacency matrix $A\in {R\;}^{n*n}$, 62-dimensional features $H_{\mathrm{handcrafted}}\in R^{n*62}$, 1280-dimensional features $H_{\mathrm{LLM}}\in R^{n*1280}$ extracted by LLM and the relative coordinates of the nodes in the 3D space $X\in R^{n*3}$, *n* is the number of nodes. The features were divided into six groups, the first one is the handcrafted features, and the second to the fifth group are four chunks uniformly divided by the large model features, and the *i*-th group is noted as $H_{{\mathrm{chunk}}_{i}}\in R^{n*320}$. The last group of features $H_{\mathrm{all}}\in R^{n*1342}$ is the concatenation of all the features, each group of features will be independently trained.

We denote the protein molecular graph as G=<V, E>, with V denoting the set of nodes and E denoting the set of edges, and use the ASAP module to extract subgraphs $G_{\mathrm{pool}}$ from G. The features and 3D coordinates of the nodes in the *l*-th layer and adjacency matrix of G and $G_{\mathrm{pool}}$ are denoted as $\left[H^{l}\in R^{n*\dim},X^{l}\in R^{n*3},\;A\in R^{n*n}\right]$ and $\left[H_{\mathrm{pool}}^{l}\in R^{pn*\dim},X_{\mathrm{pool}}^{l}{\in R}^{pn*3},A_{\mathrm{pool}}\in R^{pn*pn}\right]$, where *dim* represents feature dimension and *p* represents the sampling rate. The message passing process in each layer consists of four stages: on original graph, from original graph to subgraph, on subgraph, and from subgraph to original graph. Within each stage of layer *l*, the updating of features $H^{l}$, coordinates $X^{l}$ is done by the EGCL, denoted as Equation (1):


(1) $$H^{l},X^{l}=\mathrm{EGCL}[H^{l},X^{l},A]$$

The final output features are transformed into a two-dimensional prediction vector via a Multi-Layer Perceptron (MLP). These vectors are then concatenated in the row direction to serve as input for a meta-model that employs logistic regression. The final prediction is derived from this meta-model.

### 2.3 Proteins representation

In the handcrafted feature, we construct each protein sequence as an undirected graph G, where each node represents an amino acid in the sequence, and the existence of edges is judged by whether the Euclidean distance D of a pair of amino acids in 3D space is greater than a threshold value t, denoted by the adjacency matrix A, writing as Equation (2):


(2) Aij={1, if Dij≤t0, if Dij>t

We use both sequence information and structural information to obtain node embedding. First, in terms of sequence information, we select Position-Specific Scoring Matrix (PSSM) and Hidden Markov Model matrix (HMM), where PSSM and HMM are obtained through the PSI-BLAST (Altschul *et al.* 1997) and HHblits (Remmert *et al.* 2011) programs, respectively. For structural information, we adopt Protein Secondary Structure features (DSSP), Atomic features (AF), and pseudo-positional embedding features (PEF), with specific data shown in Table 1, available as supplementary data at *Bioinformatics* online.

In the handcrafted features section, we concatenate the 20-dimensional PSSM and HMM, the 14-dimensional DSSP, and the 7-dimensional AF with the 1-dimensional PEF to obtain the 62-dimensional nodal feature embedding shown in Equation (3):


(3) $$H_{\mathrm{handcrafted}}=[H_{\mathrm{PSSM}},H_{\mathrm{HMM}},H_{\mathrm{DSSP}},H_{AF},H_{\mathrm{PEF}}]$$

In LLM features, we choose the ESM2 protein LLM (Lin *et al.* 2023) as the encoder. ESM2 is a second-generation evolutionary scale modelling tool based on the Transformer architecture. ESM2 is pre-trained by self-supervised learning on the UniRef database containing billions of protein sequences, and is able to efficiently capture the evolutionary patterns, structural constraints, and functional relevance. Its deep attention mechanism generates context-aware amino acid residue embedding vectors. These 1280-dimensional high-order representations not only contain local structural preference information, but also encode cross-sequence long-range dependencies, providing a rich semantic representation basis for downstream tasks. By inputting each protein in the dataset into the ESM2 model, we obtained the generation of a 1280-dimensional node representation on each amino acid, denoted as $H_{\mathrm{LLM}}$.

### 2.4 Graph collapse and unpooling

Inspired by Ying *et al.* we integrate graph collapse and unpooling mechanisms at each hidden layer. During feature updating, graph collapse enables not only intra-neighborhood message passing among amino acids but also inter-cluster message propagation between local clusters through subgraphs constructed by ASAP-sampled nodes. After updating the features in the subgraph, the features of the subgraph and the original graph need to be fused. A strategy based on 0-padding and residual connection of unsampled nodes is provided in Graph U-net. However, this method does not feedback the features of the subgraph nodes into their respective neighborhoods, which gives rise to a non-negligible loss of information. To address this problem, inspired by the work of Sestak *et al.* implement feedback by unpooling. We complete the graph collapse and unpooling in four stages. The first three stages are graph collapse, and the last stage is unpooling. Each stage is composed of an independent EGCL. The adjacency matrix for each stage is as follows:

Stage 1: Update the features once on the original graph with the adjacency matrix shown in Equation (4), this phase is the same as normal EGNN. We only use the attention mechanism for the node representation H in this phase and do not use coordinate normalization or layer normalization:


(4) $$A_{\mathrm{stage}1}=A$$

Stage 2: The original graph node passes information to the subgraph node once in a unidirectional way. The node index of the subgraph is denoted as idx, edges whose end-points are not in idx and whose start-points are in idx are removed from the adjacency matrix in this stage, as shown in Equation (5):


(5) Astage2ij={1, if j∈idx and i∉idx and Aij=10, else

Stage 3: Update the features once within the subgraph nodes. This step allows for information exchange between different neighborhoods on the protein. The adjacency matrix used is the adjacency matrix of the subgraph, as shown in Equation (6):


(6) $$A_{\mathrm{stage}3}=A[\mathrm{idx},\mathrm{idx}]$$

Stage 4: The subgraph nodes feedback information to the original graph nodes.

The adjacency matrix is the transposed matrix of stage 2, as shown in Equation (7):


(7) $$A_{\mathrm{stage}4}={A_{\mathrm{stage}2}}^{T}$$

The advantage of this mechanism is that in each hidden layer, any amino acid residue can indirectly exchange information with distant nodes once through the mediation of sampling nodes, effectively alleviating the over-squashing phenomenon of graph neural networks, and avoiding the step of adding virtual edges.

### 2.5 Adaptive structure-aware pooling

**Figure 3.**
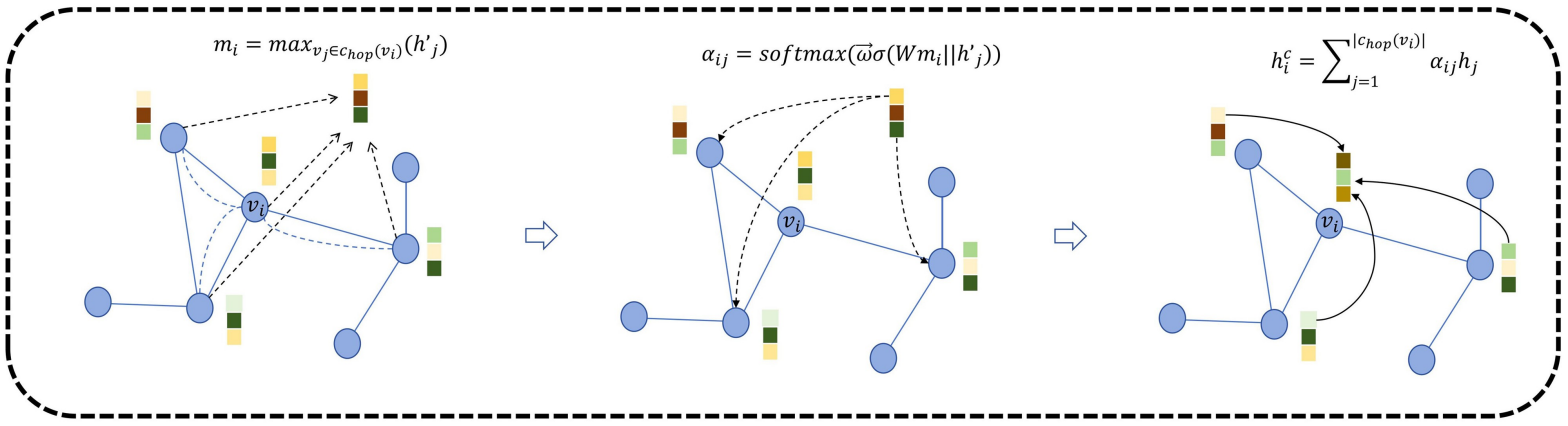
Overview of the framework of the Master to Token attention mechanism. Firstly, we obtain the main query vector of information common to the clustered clusters centered on node *i*, then we compute the affiliation of each node to cluster *i* through the node features, and finally, we compute the node features containing the clustered information based on affiliation.

The framework of ASAP is shown in Fig. 2b. ASAP first performs soft clustering of nodes by defining all nodes as the center of mass of a cluster $c_{\mathrm{hop}}(v_{i})$, hop is a hyper-parameter, and each cluster can only represent local neighbors within a radius of hop. The node features H and the adjacency matrix A are passed through an independent EGCL, and the output is notated as H′= [${h\prime }_{1}$,${h\prime }_{2}$,…,${h\prime }_{n}$]$\in R^{n*\dim}$. The M2T attention mechanism is used next, as shown in Fig. 3. First, create a main query vector $m_{i}\in R^{\dim}$. It represents the public information of all nodes in the cluster, thought to be the center. As shown in Equation (8):


(8) $$m_{i}=\max_{v_{j}\in c_{\mathrm{hop}}(v_{i})}({h^{\prime }}_{j})$$

Each element of $m_{i}$ takes the maximum of all nodes in the cluster. The additive attention mechanism is used to make m attend to all the nodes in the cluster, and the affiliation of node $v_{j}$ is calculated for the cluster $c_{h}(v_{i})$ with $v_{i}$ as the center of mass as shown in Equation (9), where ${\vec{\omega }}^{T}\in R^{\dim}$ and ***W***$\in R^{\dim*1}$ are the learnable weight matrices:


(9) $$\alpha_{ij}=\mathrm{softmax}({\vec{\omega }}^{T}\sigma (Wm_{i}||{h^{\prime }}_{j}))$$

The node features containing clustering information are obtained based on $\alpha_{ij}$ as shown in Equation (10):


(10) $$h_{i}^{c}=\sum_{j=1}^{\left|c_{\mathrm{hop}}(v_{i})\right|}\alpha_{ij}h_{j}$$

This set of features is used to score the nodes instead of H. For the scoring function we use a special graph convolution layer: the Local Extreme Convolution (LEConv), which is a graph convolution method that can capture the local extreme value information. The output is the score of each node as shown in Equation (11), where $N_{i}$ denotes the neighbourhood of node *i*, $W_{1}{,W}_{2},W_{3}\in R^{\dim*\dim}$ are the learnable parameter matrix and σ is the activation function:


(11) $$\phi_{i}=\sigma (h_{i}^{c}W_{1}+\sum_{j\in N_{i}}A_{ij}(h_{i}^{c}W_{2}-h_{j}^{c}W_{3}))$$

The final score vector of the node $\Phi =[\phi_{1},\phi_{2},\ldots ,\phi_{n}]\in R^{n}$ is obtained. Then select the top of these scores to generate the index idx. Obtain node features $H_{\mathrm{pool}}$ and adjacency matrix $A_{\mathrm{pool}}$ based on indexing, as shown in Equations (12)–(13), where ⊙ denotes the Hadamard product, this step makes the parameters on the ASAP module learnable.


(12) $$H_{\mathrm{pool}}=(H⊙\Phi )(\mathrm{idx},:)$$

(13) Apoolij={1, if i∈idx and j∈idx0, if i∉idx or j∉idx

As the hub of graph collapse, sampled nodes need to balance the completeness of the information in the represented neighborhood with the criticality from a global perspective. Topkpooling in Graph U-net scores the nodes by comparing the magnitude of all the node features on the projection vectors, and SAGpooling (Lee *et al.* 2019) computes them through another convolutional layer. Compared to methods such as Topkpooling and SAGpooling, ASAP’s scoring strategy improves the clustering problem. Due to the high tendency of protein graphs to be homologous, node embeddings of neighboring amino acids tend to show similarity. In the strategy of the first two approaches, when a node’s score is high, it significantly increases the scores of neighboring nodes, leading to a tendency for sampling operations to select local clusters of a particular amino acid rather than clusters that represent the entire protein. While ASAP mitigates the clustering problem by first soft clustering the nodes and considering the effects inside and outside the clustered clusters at the same time when scoring. At the same time, this strategy prefers to keep the graph connected, when the size of the protein graph tends to infinity, the minimum pooling rate of the ASAP method to keep the constructed subgraphs connected tends to 50% while Topkpooling and SAGpooling tends to 100%, so in order to keep the connectivity at even lower pooling rates, it is usually necessary to add virtual edges.

### 2.6 *E*(*n*) equivariant graph convolutional layer

*E*(*n*) equivariant graph neural networks are dedicated to the modelling of graph-structured data with geometric symmetry, which maintains the equivariant properties of the model by dynamically updating the features and spatial coordinates of nodes. The core component of this network architecture is EGCL. EGCL synchronously updates the node representations and their spatial coordinates in the current layer by aggregating node coordinates with feature information. As shown in Fig. 2c, compared to traditional graph convolutional neural networks that focus only on the processing mechanism of node feature updating, the innovation of EGCL lies in the embedding of the coordinate updating operator into the feature aggregation process, implementing a message passing mechanism that satisfies the E(n) group equivalence constraints. An easy proof of equivariance is provided in the Supplementary Materials, available as supplementary data at *Bioinformatics* online. As shown in Equations (14)–(17), this layer coordinates the processing of geometric relations and semantic features among nodes through learnable equivariant functions, which strictly satisfy the equivariance requirements under translation and rotation while maintaining the coupling of coordinate update and feature transformation.


(14) $${\mathrm{mp}}_{ij}^{l}=\varphi_{e}(h_{i}^{l},h_{j}^{l},{\left|\left|x_{i}^{l}-x_{j}^{l}\right|\right|}^{2},a_{ij})$$

(15) $$X_{i}^{l+1}=x_{i}^{l}+C\sum_{j\neq i}\left(x_{i}^{l}-x_{j}^{l}\right)\varphi_{x}({\mathrm{mp}}_{ij}^{l})$$

(16) $${\mathrm{mp}}_{i}^{l}=C\sum_{j\neq i}\left({\mathrm{mp}}_{ij}^{l}\right)$$

(17) $$h_{i}^{l+1}={h_{i}^{l}+\varphi }_{h}({h_{i}^{l},\mathrm{mp}}_{i}^{l})$$

In the above equation ${\mathrm{mp}}_{ij}^{l}$ denotes the message passed between node *i* and node *j* at layer *l*. We add the relative squared distance ${\left||x_{i}^{l}-x_{j}^{l}|\right|}^{2}$ between the two coordinates to the message aggregation of the edge. The coordinates of all nodes are subsequently updated as a vector field in the radial direction, with each position updated by a weighted sum of the relative differences of all coordinates. The weights are calculated by$\;m_{ij}^{l}$*. C* is set as $\frac{1}{N-1}$, *N* is the number of neighborhood nodes.

Finally, as in Equation (17), the information ${\mathrm{mp}}_{i}^{l}$ received by node *i* at layer *l* is expressed as a sum of the information of all its neighbors, which is combined with $h_{i}^{l}$ to obtain the updated features $h_{i}^{l+1}$. The $\varphi_{e}$,$\varphi_{x}$ and $\varphi_{h}$ mentioned above denote the update functions for edges, coordinates, and features, respectively, and they all consist of multiple MLPs with activation functions. In addition, to mitigate the over-smoothing problem, as shown in Equation (18), we include residual connections between each layer of EGCL:


(18) $$h^{l+1}=(h^{l}+\mathrm{EGCL}[h^{l},x^{l},A])$$

### 2.7 Ensemble model based on bagging and meta-model

In order to further improve the robustness of the model, we augment the data with the ESM2 protein LLM, and at the same time, in order to avoid dimensionality catastrophe, we divide this set of features into four groups of 320-dimensional features based on the Bagging (Bootstrap Aggregating) integration strategy as shown in Equation (19).


(19) $${\mathrm{chunk}}_{i}=H_{\mathrm{ESM}}[:,(i-1)*320:i*320]$$

After that with the features of each chunk as input, we train four models of the ASCE framework. Finally, we train another model with the 62-dimensional handcrafted feature chunks as inputs, and concatenate the prediction result of the five models with the binary prediction result of ASCE-PPIS as input to train the meta-model of the logistic regression framework as shown in Equations (20)–(21):


(20) $$\hat{x}=[y_{0},y_{1},y_{2},y_{3},y_{4},y_{5}]$$

(21) $$\hat{y}=\mathrm{LogisticRegression}(\hat{x})$$

The output of the meta-model is the final prediction, indicating whether this node is an interaction site or not.

## 3 Results and discussion

### 3.1 Evaluation metrics

The main measures of model performance are AUROC and AUPRC, which are used to select appropriate features and hyperparameters for the model. More details of the remaining evaluation metrics can be found in the Supplementary Materials, available as supplementary data at *Bioinformatics* online.

The AUROC metric focuses on the overall performance of the model at different thresholds and assesses the model’s ability to discriminate between positive and negative samples by calculating the area under the subject’s work characteristic curve. The ROC curve takes the false positive rate (FPR) as the horizontal coordinate, and the recall rate (TPR) as the vertical coordinate. The closer the value of AUROC is to 1, the better the model’s classification performance is, and it is suitable for assessing various types of classification tasks.

AUPRC is concerned with the predictive performance of the model on positive samples and assesses the model’s ability to recognize positive samples by calculating the area under the precision-recall curve. The AUPRC metric is highly informative in the case of unbalanced datasets, as it effectively assesses the model’s ability to recognize a small number of classes.

### 3.2 Experiment details

We perform 5-fold cross-validation on Train334. The final hyperparameters of the model partly refer to the findings in AGAT-PPIS, and are shown in Table 2.

**Table 2.** Hyperparameter of the experiment.

Distance thresholds	14 Å
Rseudo-position embedding of residues	15 Å
Number of layers	8
Sampling rate	30%
Hidden dims	128
Learing rate	5 × 10^–4^
Regularization parameter	1 × 10^–5^

The model is optimized using a cross-entropy loss function and Adam optimizer, each training contains 60 epochs; a learning rate decay strategy is used during training, when the AUPRC value does not increase for 10 consecutive epochs, the learning rate will be reduced to 0.6 times, down to a minimum of 10^−6^; cross-entropy loss is used to guide the gradient update.

### 3.3 Performance comparison with other methods

The performance of ASCE-PPIS on Test60 (Take the best of 5 folds) was compared with six protein sequence-based PPIS prediction methods, six graph-based methods. The protein sequence-based methods include: PSIVER, ProNA2020 (Qiu *et al.* 2020), SCRIBER (Zhang and Kurgan 2019), DeepPPISP, DLPred, DELPHI, and EnsemPPIS. The graph-based methods include: Graph-PPIS, AGAT-PPIS, GHGPR-PPIS, E(Q)AGNN-PPIS, MEG-PPIS, and AGF-PPIS. The experimental results are shown in Table 3.

**Table 3.** Performance comparison with other models on Test60.^a^

Method	ACC	Precision	Recall	F1	AUROC	AUPRC	MCC
PSIVER	0.561	0.188	0.534	0.278	0.573	0.190	0.074
ProNA2020	0.738	0.275	0.402	0.326	N/A	N/A	0.176
SCRIBER	0.667	0.253	0.568	0.350	0.665	0.278	0.193
DeepPPISP	0.657	0.243	0.539	0.335	0.653	0.276	0.167
DLPred	0.682	0.264	0.565	0.360	0.677	0.294	0.208
DELPHI	0.697	0.276	0.568	0.372	0.699	0.319	0.225
EnsemPPIS	0.732	0.375	0.532	0.440	0.719	0.405	0.277
Graph-PPIS	0.776	0.368	0.584	0.451	0.786	0.429	0.333
AGAT-PPIS	0.856	0.539	0.603	0.569	0.867	0.574	0.484
GHGPR-PPIS	0.860	0.551	0.620	0.583	0.869	0.596	0.501
E(Q)AGNN-PPIS	0.870	0.580	0.680	0.620	N/A	0.650	0.550
MEG-PPIS	0.878	0.605	**0.657**	0.630	0.892	0.666	0.558
AGF-PPIS	0.860	0.551	0.620	0.584	0.870	0.599	0.501
ASCE-PPIS (Ours)	**0.897**	**0.678**	0.653	**0.666**	**0.921**	**0.734**	**0.605**

a The results of the comparison method are taken from the corresponding citations. N/A indicates that there is no available data. The best results are in bold.

The data shows that ASCE-PPIS outperforms existing methods in all metrics on the benchmark dataset Test60. Non-GNN models lack the full use of protein molecular structure information, while existing graph-based methods represented by AGAT-PPIS, due to the requirement of invariance to translation and rotation, cannot process the equivariant 3D coordinates in vector form and can only convert them into scalar forms such as distance and angle, thereby limiting the ability to learn directional interactions. Our method significantly improves the performance with an increase of 0.019 on ACC, 0.036 on F1 scores, 0.029 on AUROC, 0.047 on MCC, and 0.068 on AUPRC, compared to MEG-PPIS. In order to further validate the robustness of ASCE-PPIS, we also compare their performance on independent test sets Test315-28 and UBtest31-6 with these methods, and the experimental results are shown in Table 4. The results show that the method has good generalization and exhibits remarkable performance on multiple datasets.

**Table 4.** Performance comparison of ASCE-PPIS and other methods on the Test315-28 and UBtest31-6.^a^

Method	Test315-28		UBtest31-6	
	MCC	AUPRC	MCC	AUPRC
DeepPPISP	0.169	0.256	0.162	0.217
Graph-PPIS	0.349	0.423	0.313	0.339
AGAT-PPIS	0.481	0.572	0.327	0.365
GHGPR-PPIS	0.486	0.564	0.356	0.367
MEG-PPIS	**0.557**	**0.651**	0.356	0.396
E(Q)AGNN-PPIS	0.497	0.604	0.362	0.421
AGF-PPIS	0.484	0.565	0.378	0.397
ASCE-PPIS (Ours)	0.550	0.641	**0.410**	**0.493**

a The results of the comparison method are taken from the corresponding citations. The best results are in bold.

## 4 Ablation experiment

### 4.1 Model structure ablation experiment

**Figure 4.**
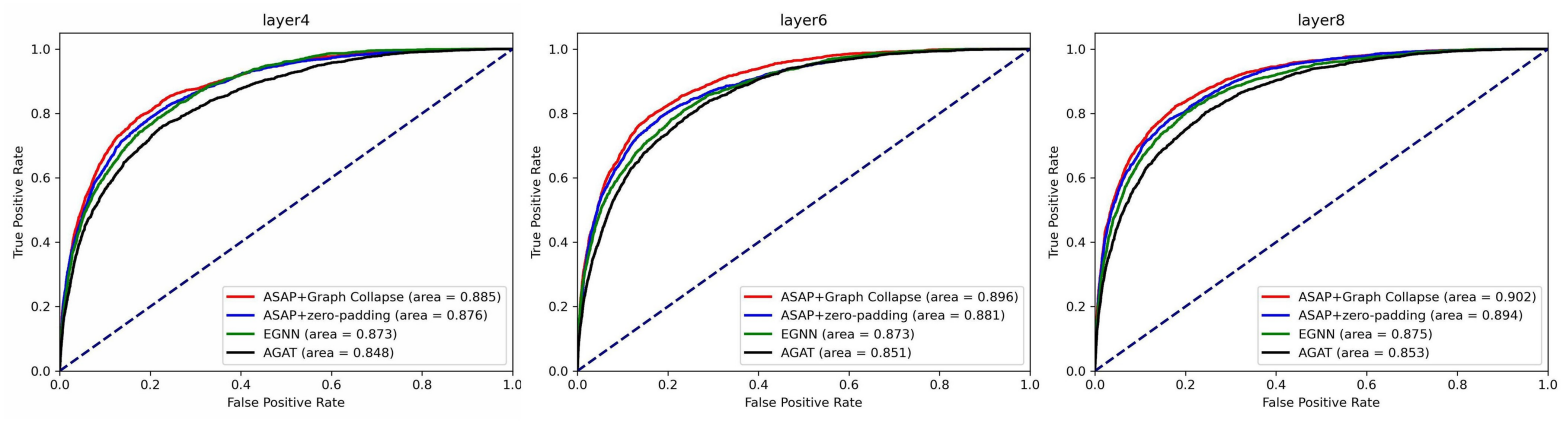
Performance comparison of models with and without the ASAP module and graph collapse at different hidden layer depths.

In order to verify the impact of each module on the model performance, we compare the performance of the benchmark model AGAT trained with handcrafted features compared to the three models using only EGNN, using ASAP with 0-padding and ASAP with graph collapsing on Test60 under different number of layers, and the ROC curves of each model are shown in Fig. 4. The experimental results show that the multi-scale features provided by ASAP enhance the model performance at different layer numbers, and the graph collapse mechanism always outperforms 0-padding. In addition, when AGAT and EGNN saturated their performance with increasing network depth due to over-squashing, the ASCE model still maintains the performance gain. This phenomenon verifies that ASAP with graph collapse effectively mitigates the representation degradation problem of deep graph neural networks by adjusting the receptive field and indirect communication. The impact of our method on the high-layer model can be more intuitively observed through T-SNE, and the relevant results can be found in the Supplementary Material, available as supplementary data at *Bioinformatics* online.

### 4.2 Comparison of different sampling rate

**Figure 5.**
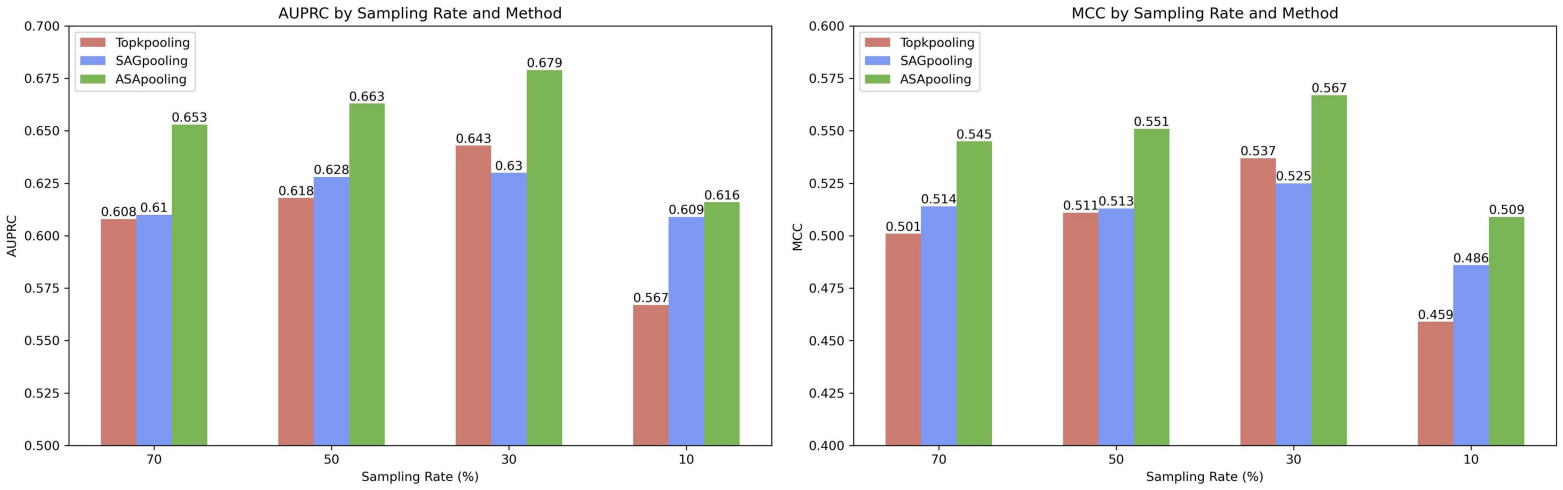
Comparison of AUPRC and MCC metrics of the model after sampling nodes with three methods, Topkpooling, SAGpooling, and ASApooling, at different sampling rates.

This experiment compares the impact of three sampling methods, Topkpooling, SAGpooling and ASAP, on the performance of a single model based on handcrafted features at different sampling rates (70%, 50%, 30% and 10%) on the Test60 dataset, and the results are shown in Fig. 5. Experimental results show that all three methods achieve optimal AUPRC and MCC metrics at 30% sampling rate. Notably, when the sampling rate is lower than 30%, the model performance is significantly degraded due to the impaired topological integrity of the subgraph as a result of excessive sparsification. Further analysis reveals that Topkpooling and SAGpooling are limited by local clustering effects, and their node selection mechanism based on neighborhood similarity is difficult to take into account the global characteristics of the protein structure. While ASAP achieves precise localization of key nodes through two attention scores, intra-cluster and global, after soft clustering, and thus exhibits better performance at different sampling rates.

### 4.3 Comparison of different hops

This experiment demonstrates the impact of the hop of ASAP on the model’s performance. We compared the performance of the ASCE-PPIS model, trained with handcrafted features, on the Test60 dataset when hop values were set to 1, 2, and 3. The result is shown in Table 5.

**Table 5.** Performance of different hops.^a^

hop	F1	AUROC	AUPRC	MCC
1	**0.637**	**0.902**	**0.679**	**0.566**
2	0.633	0.897	0.669	0.563
3	0.626	0.895	0.656	0.551

^a^ The best results are in bold.

Theoretically, increasing the hop enhances the potential rationality of the sampling process. However, due to the inherent density of protein graphs, a higher hop leads to an exponential increase in the number of sampled nodes. Consequently, the receptive fields of different sites become increasingly similar, exhibiting an effect analogous to over-smoothing in graph neural networks. Our experiments show that the hop has little effect on overall performance, but it significantly increases computational time complexity. Therefore, based on our experimental results, we recommend setting the hop value to 1.

### 4.4 Sub-models and ensemble strategies

**Figure 6.**
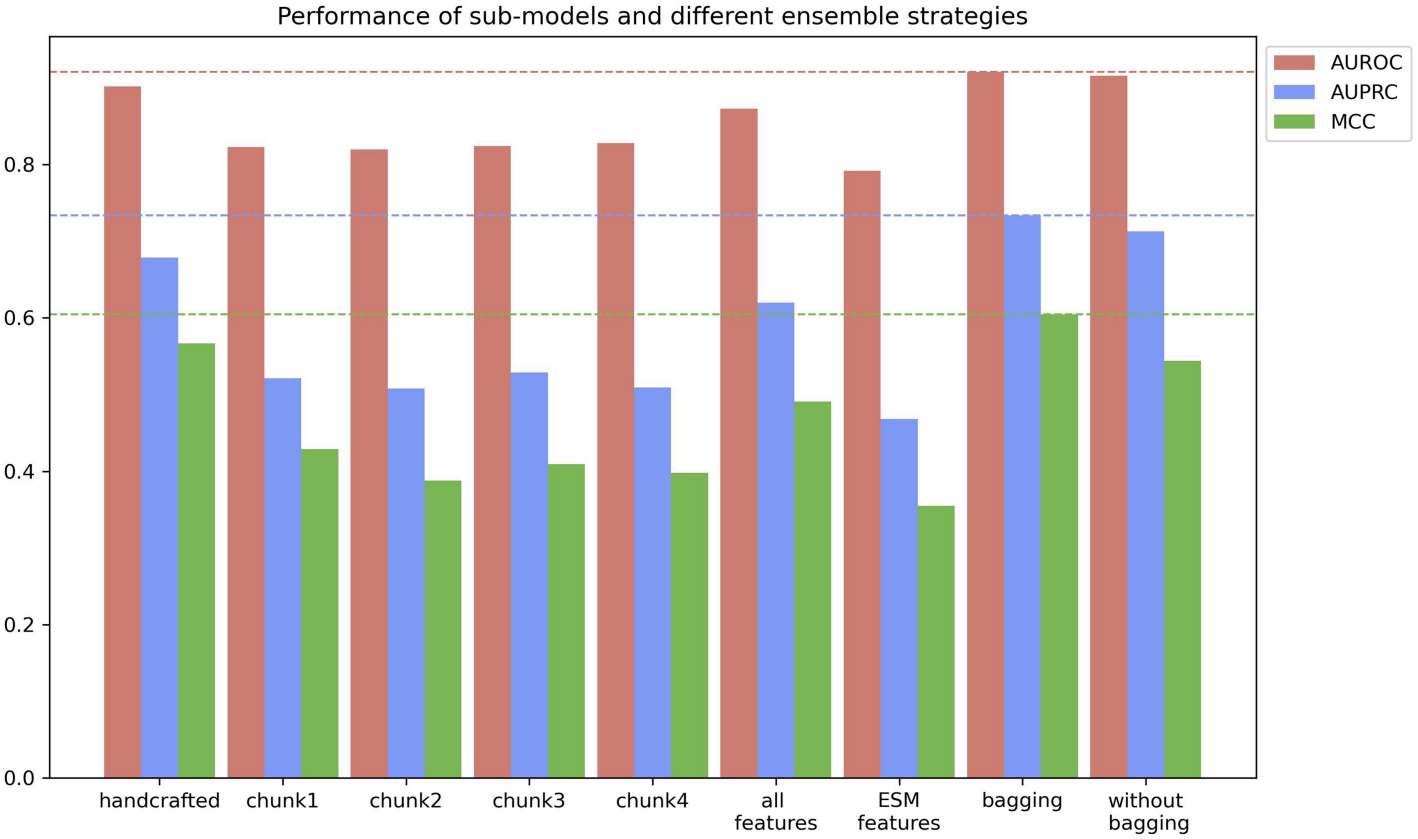
Performance of models from seven groups of features and two ensemble models with or without bagging.

In this section, we analyze the role of ensemble models versus bagging. The experimental results are shown in Fig. 6 (More details can be found in Table 2, available as supplementary data at *Bioinformatics* online). The nine sets of data are model trained on handcrafted features, four chunks of LLM features, all features spliced together, LLM features only, and two ensemble models with or without using bagging.

The result shows that the fusion of large model features with hand-crafted features through an ensemble learning framework significantly improves performance compared to a single model, revealing the complementary advantages between different features, and the multi-perspective feature fusion can effectively break through the characterization bottleneck of a single model and provide more robust information for protein function prediction. Notably, models trained directly with ESM features perform inferiorly to models from each chunk. The work of Luo Z *et al.* (Luo *et al.* 2024) indicates that directly using features from LLMs for downstream tasks leads to feature redundancy, which increases the risk of overfitting. Therefore, dimensionality reduction is necessary for these features. Dividing the 1280-dimensional vector into 4 chunks serves as a simple form of linear dimensionality reduction, thereby mitigating the potential issue of feature redundancy. On the other hand, since ESM2 uses multi-head attention during training, each attention head focuses on different aspects. Therefore, each chunk is essentially a combination of outputs from several attention heads, and thus contains different semantic information. We also performed t-SNE on the four chunks, and the results can be found in the Fig. 1, available as supplementary data at *Bioinformatics* online. The Bagging strategy acts as a multi-perspective integration for models trained on different features, making the prediction results less sensitive to noise in the data, thereby improving the overall performance of the ensemble model.

## 5 Case study

**Figure 7.**
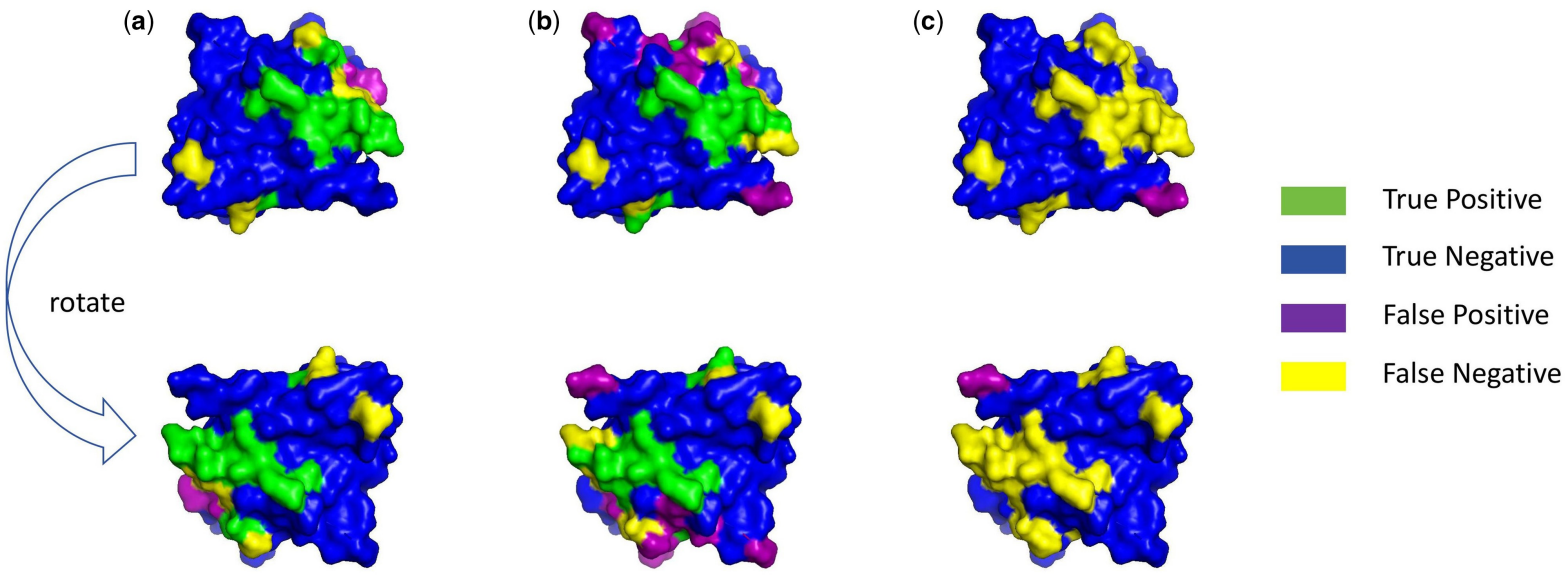
An example case of protein ‘5dmrA’; (a) interaction site predicted by ASCE-PPIS; (b) interaction site predicted by GHGPR-PPIS; (c) interaction site predicted by AGAT-PPIS.

We conducted a specific case study to evaluate the predictive ability of the ASCE-PPIS model for specific protein chains. Table 6 shows the PPIS prediction results for protein 5dmr chain A by our model and the GHGPR-PPIS model and the AGAT-PPIS model. The results show that our model predicts more true positive sites and true negative sites, as well as fewer false positive and false negative sites compared to the GHGPR-PPIS model. As shown in Fig. 7, we also visualized the prediction results using the PyMOL program. By looking at the colored sections, we can find that ASCE-PPIS has significantly fewer associated false positive sites in the prediction results, indicating that our model can better identify PPIS.

**Table 6.** Prediction results of ASCE-PPIS, GHGPR-PPIS, and AGAT-PPIS on specific proteins.

Model	TP	TN	FP	FN
ASCE-PPIS	13	111	4	11
GHGPR-PPIS	12	87	28	12
AGAT-PPIS	2	96	19	22

## 6 Features importance

**Figure 8.**
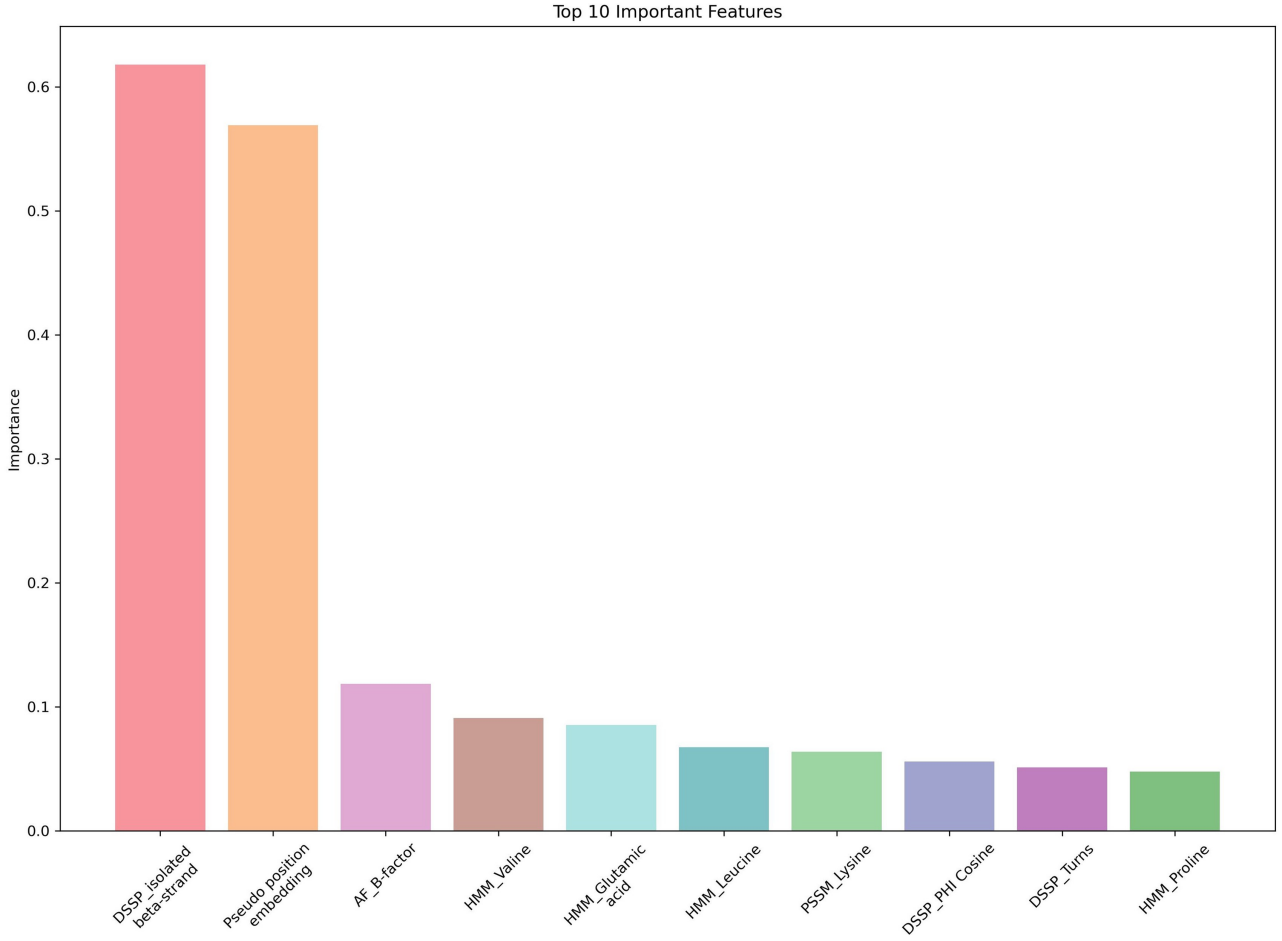
Feature importance calculated by GraphLIME, with higher values indicating higher bias in model predictions when this data is perturbed.

To improve the credibility of the model, we analyze the importance of the 62 + 1280-dimensional features. Compared to ablation experiments that enumerate feature combinations in similar work, we perform a more efficient and intuitive feature importance analysis with the help of the interpretable algorithm GraphLIME (Huang *et al.* 2023). LIME (Ribeiro *et al.* 2016) is an interpretable method based on perturbation and agent modeling by generating a new set of data at local perturbation and using this set of data to train highly interpretable agent models to account for the importance of each feature. GraphLIME is an extension of this algorithm for GNNs so that the perturbation can take into account topology. GraphLIME generates the importance of each feature one at a time for each amino acid on a sequence, we run it once for each amino acid on the protein sequences in Test60, Test315-28, UBTest31-6, and sum and normalize the results to obtain the importance of the feature over all samples, sum and normalize the results to obtain the importance of the feature over all samples. Setting the sampling range as 2 hops, the top 10 important features of the output are shown in Fig. 8 (more features can be found in Table 3, available as supplementary data at *Bioinformatics* online). The three most prominent of these features are whether the structure is an isolated beta-strand morphology, pseudo-positional embedding, and the B-factor of the atom. This information can be interpreted from a biological perspective.

The work of Arenas *et al.* (Arenas *et al.* 2019) revealed a correlation between the beta-folded structure and the protein interaction site. In β-folding, the structure is stabilized by the formation of hydrogen bonds between the chains through the N-H and C = O groups of the main chain. Isolated β-chains, on the other hand, are usually unable to form such hydrogen bonds and may rely on interactions with surrounding amino acid side chains, water molecules, or ring regions to maintain stability and are therefore prone to forming interaction sites. From a biological perspective, pseudo-position embeddings are part of structural information, related to mechanisms such as spatial accessibility and physicochemical interactions, providing a physical basis for PPIS prediction. Furthermore, from a machine learning perspective, pseudo-position embeddings are distance-based representations that enable the model to be invariant to translational and rotational transformations in scalar features. B-factor is a key parameter for understanding the dynamic behavior of proteins; high B-factor regions of target proteins may suggest potential variant sites or binding pockets in drug design. The above conclusions demonstrate that the prediction logic of the model is consistent with the a priori knowledge of biology, reflecting the reliability of the method.

## 7 Conclusion

In this paper, we propose a new method, ASCE-PPIS, for predicting protein–protein interaction sites. In ASCE-PPIS, we apply structure-aware pooling and graph collapse on equivariant graph neural networks and integrate large model features with handcrafted features through an ensemble model. We trained the model on the Train334 dataset and achieved good results on three independent test sets, with significantly better performance compared to Graph-PPIS, AGAT-PPIS, and GHGPR-PPIS, which also use graph neural networks. We propose to enhance the model representation with multi-scale features and mitigate the over-squashing problem by graph collapse. Based on ablation experiments, the module effectively improves the accuracy, AUROC, and AUPRC. In addition, we analyzed the feature importance by an interpretable algorithm and proved that the predictions of the model were consistent with biological knowledge, validating the effectiveness of the method. However, our model still has some limitations. First, our model is still a black box model in terms of its prediction process, although it can be explained with the help of interpretable algorithms. Second, we did not effectively utilize the edge information on the graph. In our future work, we will conduct in-depth research to solve these problems.

## Supplementary Material

btaf423_Supplementary_Data

## Data Availability

The data and code are available at https://github.com/nunhehheh/ASCE-PPIS.
