# Supplementary material for: ASCE-PPIS: a protein–protein interaction sites predictor based on equivariant graph neural network with fusion of structure-aware pooling and graph collapse
Source: Bioinformatics. 2025 Jul 24;41(8):btaf423. doi: 10.1093/bioinformatics/btaf423 (PMC12342974; doi:10.1093/bioinformatics/btaf423)
Supplement: btaf423_Supplementary_Data [file btaf423_supplementary_data.doc]

**Supplementary**

**Details of handcrafted features**

| **Table S1**. The statistics of features | | |
| --- | --- | --- |
| Name | Dimension | Description |
| PSSM | 20 | The probability of occurrence of different amino acids at each position in the sequence |
| HMM | 20 | The process of generating a sequence of observations from a hidden state |
| DSSP | 14 | Secondary structure information |
| AF | 7 | Atomic features |
| PEF | 1 | Euclidean distance of all nodes from the first residue coordinates |

The amino acids corresponding to each bit of the PSSM and HMM are Glycine, Alanine, Valine, Leucine, Isoleucine, Serine, Threonine, Aspartic acid, Glutamic acid, Asparagine, Glutamine, Lysine, Arginine, Proline, Phenylalanine, Tyrosine, Tryptophan, Histidine, Methionine and Cysteine. The first 9 dimensions of the DSSP are the secondary structure represented by one-hot encoding, and the last 5 dimensions represent the sine/cosine values of the peptide backbones PHI and PSI and the relative solvent-accessible surface area. The atomic features include atomic mass, B-factor, whether it is a residue side-chain atom, electronic charge, number of hydrogen atoms bonded to it, whether it is in a ring and the van der Waals radius of that atom.

**Equivariance Proof**

The EGNN is characterized by the equivariance of the updated node coordinates in each layer，the same result will be obtained for the vectorial features of the atoms by transforming then updating and updating then transforming as shown in Equation (1):

$h^{l+1},Qx^{l+1}+g=EGCL(h^{l},Qx^{l}+g)$ (1)

Q denotes rotational transformation and g denotes translational transformation. An easy proof of equivariance is provided in the supplementary materials.

The update for node coordinates in the EGCL layer is shown in Equation (2):

$\begin{aligned} X_{i}^{l+1}=x_{i}^{l}+C\sum_{j\neq i} \left( x_{i}^{l}-x_{j}^{l} \right)\varphi_{x}\left( m_{ij}^{l} \right) \end{aligned}$ (2)

We note that if a transformation is applied to x, as shown in Equation (3):

$Qx_{i}^{l}+g+C\sum_{j\neq i} \left( Qx_{i}^{l}+g-Qx_{j}^{l}-g \right)\varphi_{x}\left( m_{ij} \right)$

$=Qx_{i}^{l}+g+QC\sum_{j\neq i} \left( x_{i}^{l}-x_{j}^{l} \right)\varphi_{x}\left( m_{ij} \right)$

$=Q\left( x_{i}^{l}+C\sum_{j\neq i} \left( x_{i}^{l}-x_{j}^{l} \right)\varphi_{x}\left( m_{ij} \right) \right)+g$

$=Qx_{i}^{l+1}+g$ (3)

Thus in each layer of the EGCL, the vector features can remain equivariant.

**Evaluation metrics**

In addition to the AUROC and AUPRC mentioned in the main text, we use several other evaluation metrics to judge the model's binary discrimination ability, including Accuracy (ACC), Precision, Recall, F1-score (F1), Matthews correlation coefficient (MCC). As shown in Equation (4) to (8), TP, TN, FP and FN denote the number of true positives, true negatives, false positives and false negatives respectively.

$ACC=\frac{TP+TN}{TP+FN+TN+FP}$ (4)

$Precision=\frac{TP}{TP+FP}$ (5)

$Recall=\frac{TP}{TP+FN}$ (6)

$F1=2*\frac{Precision*Recall}{Precision+Recall}$ (7)

$MCC=\frac{TP*TN-FP*FN}{\sqrt{(TP+FP)*(TP+FN)*(TN+FP)*(TN+FN)}}$ (8)

**Dimensionality reduction of four chunks**

**
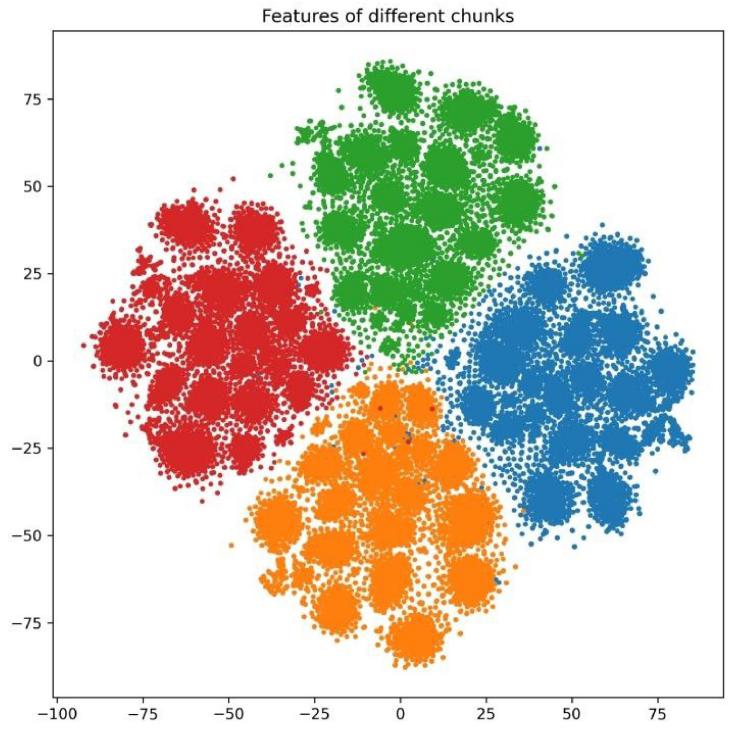
**

**Figure S1**. Results of the four chunks by t-SNE

We visualized the features of the four chunks using t-SNE, and the results are shown in Figure S2. From the figure, it can be found that the features from the four chunks are clearly separable, indicating that there are significant differences in their data distribution, which demonstrates that they can be associated with different semantic information..

**Analysis of the effectiveness**

**
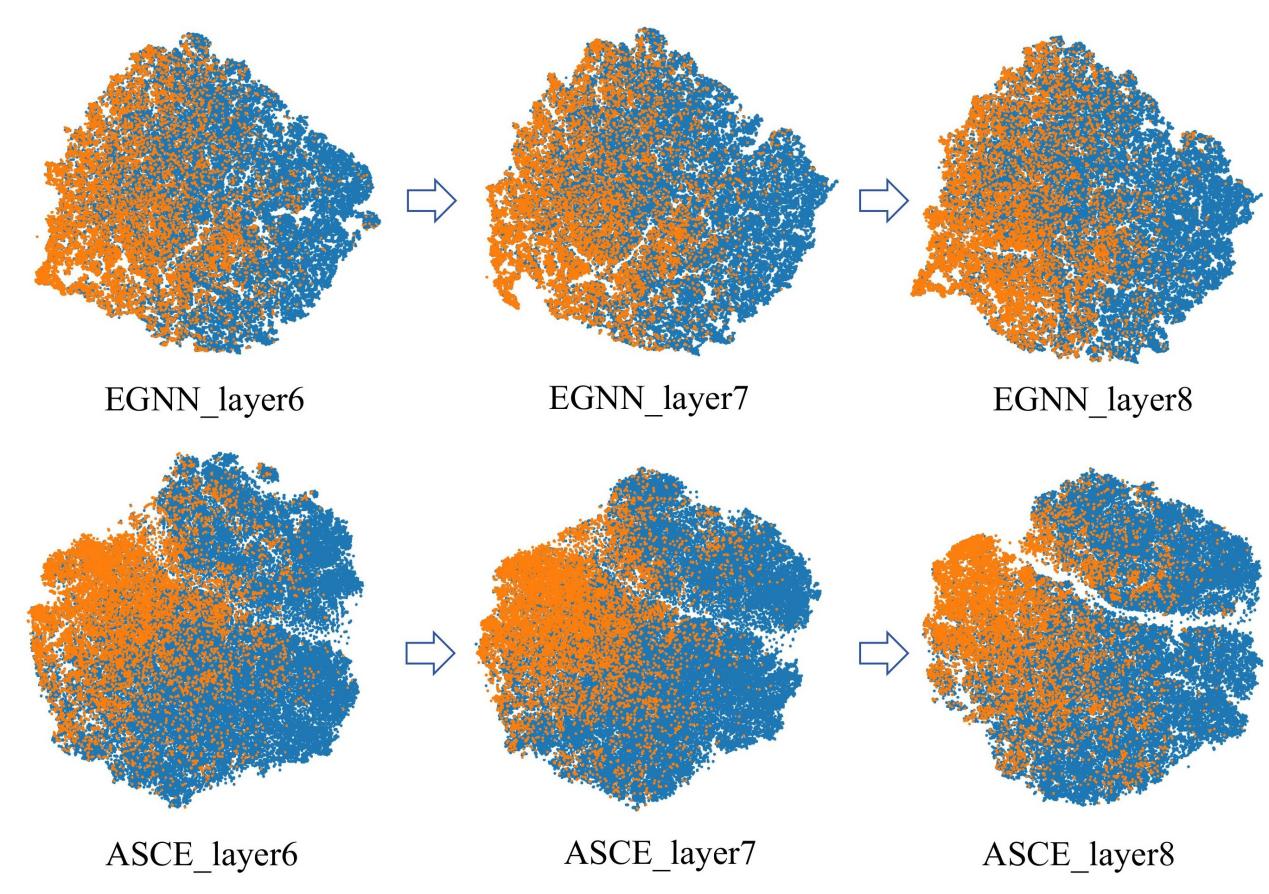
**

**Figure S2.** Results of EGNN and handcrafted feature-based ASCE in last 3 layers by T-SNE.

We visualize part of the prediction process of the model. We visualized the extracted features for the last three layers of EGNN and ASCE on Test60, Test315-28, UBTest31-6 trained on handcrafted features with T-SNE. The specific results are shown in Figure S2, where orange dots denote interaction sites and blue denotes non-interaction sites.

Experimental observations show that the feature distribution of the traditional EGNN model in the last three layers exhibits obvious convergence stagnation. Despite extending the message delivery distance by increasing the network depth, the model still fails to effectively capture the feature information of long-distance nodes.

In contrast, the visualization results of our proposed model clearly demonstrate the model's ability to capture the deep information of protein sequences by early fusion of features from the last three layers of the 8-layer network. This progressive feature aggregation process demonstrates that ASCE-PPIS is able to break through the characterization bottleneck of traditional graphical neural networks and achieve effective long-range dependency modeling.

**Detailed data of sub-models and ensemble models**

| **Table S2.**  Performance of different features | | | | | | | | | |
| --- | --- | --- | --- | --- | --- | --- | --- | --- | --- |
| Method | Test60 | | | Test315-28 | | | UBtest31-6 | | |
|  | MCC | AUPRC | AUROC | MCC | AUPRC | AUROC | MCC | AUPRC | AUROC |
| handcrafted | 0.567 | 0.679 | 0.902 | 0.547 | 0.626 | 0.898 | 0.360 | 0.411 | 0.811 |
| $\mathrm{chunk}_{1}$ | 0.429 | 0.521 | 0.823 | 0.353 | 0.427 | 0.795 | 0.417 | 0.471 | 0.831 |
| $\mathrm{chunk}_{2}$ | 0.388 | 0.508 | 0.820 | 0.374 | 0.446 | 0.817 | 0.371 | 0.417 | 0.822 |
| $\mathrm{chunk}_{3}$ | 0.409 | 0.529 | 0.824 | 0.370 | 0.441 | 0.805 | 0.398 | 0.473 | 0.829 |
| $\mathrm{chunk}_{4}$ | 0.398 | 0.509 | 0.828 | 0.359 | 0.430 | 0.801 | 0.370 | 0.417 | 0.824 |
| All features | 0.491 | 0.620 | 0.873 | 0.456 | 0.546 | 0.865 | **0.419** | **0.495** | 0.850 |
| LLM features | 0.355 | 0.468 | 0.792 | 0.313 | 0.380 | 0.779 | 0.383 | 0.437 | 0.828 |
| Ensemble Model with bagging | **0.605** | **0.734** | **0.921** | **0.550** | **0.641** | **0.902** | 0.410 | 0.493 | **0.851** |
| Ensemble Model without bagging | 0.544 | 0.713 | 0.916 | 0.541 | 0.627 | 0.890 | 0.413 | 0.488 | 0.839 |

**Feature Importance**

| **Table S3**. Features with importance greater than 0.04 | | | | | |
| --- | --- | --- | --- | --- | --- |
| Index | Feature name | Importance | Index | Feature name | Importance |
| 1 | DSSP_isolated beta-strand | 0.61807 | 11 | PSSM_Leucine | 0.04708 |
| 2 | Pseudo position embedding | 0.56907 | 12 | HMM_Lysine | 0.04651 |
| 3 | AF_B-factor | 0.11868 | 13 | PSSM_Threonine | 0.04542 |
| 4 | HMM_Valine | 0.09111 | 14 | LLM_425 | 0.04347 |
| 5 | HMM_Glutamic acid | 0.08538 | 15 | LLM_134 | 0.04325 |
| 6 | HMM_Leucine | 0.06739 | 16 | PSSM_Glutamic acid | 0.04312 |
| 7 | PSSM_Lysine | 0.06389 | 17 | LLM_755 | 0.04286 |
| 8 | DSSP_PHI Cosine | 0.05604 | 18 | DSSP_PSI Sine | 0.04118 |
| 9 | DSSP_Turns | 0.05116 | 19 | PSSM_Valine | 0.04074 |
| 10 | HMM_Proline | 0.04767 | 20 | PSSM_Serine | 0.04029 |

There are 20 features with importance greater than 0.04, as shown in table S3.
